# Dynamic Analysis of the Coupling Coordination Relationship between Urbanization and Water Resource Security and Its Obstacle Factor

**DOI:** 10.3390/ijerph16234765

**Published:** 2019-11-28

**Authors:** Kaize Zhang, Juqin Shen, Ran He, Bihang Fan, Han Han

**Affiliations:** 1 Business School, Hohai University, Nanjing 211100, China; kzzhang@hhu.edu.cn (K.Z.); jqshen@hhu.edu.cn (J.S.); 170212070002@hhu.edu.cn (H.H.); 2 Department of Ecosystem Science and Management, The Pennsylvania State University, State College, PA 16802, USA; 3 State Key Laboratory of Hydraulics and Mountain River Engineering, Sichuan University, Chengdu 610065, China

**Keywords:** urbanization, water resource security, improved coupling coordination model, obstacle degree model, Beijing

## Abstract

Water resource security is an important condition for socio-economic development. Recently, the process of urbanization brings increasing pressures on water resources. Thus, a good understanding of harmonious development of urbanization and water resource security (WRS) systems is necessary. This paper examined the coordination state between urbanization and WRS and its obstacle factors in Beijing city, utilizing the improved coupling coordination degree (ICCD) model, obstacle degree model, and indicator data from 2008 to 2017. Results indicated that: (1) The coupling coordination degree between WRS and urbanization displayed an overall upward tendency during the 2008–2017 period; the coupling coordination state has changed from an imbalanced state into a good coordination state, experiencing from a high-speed development stage (2008–2010), through a steady growth stage (2010–2014), towards a low-speed growth (2014–2017). (2) In urbanization system, both the social and spatial urbanizations have the greatest obstruction to the development of urbanization-WRS system. The subsystems of pressure and state are the domain obstacle subsystems in WRS system. These results can provide important support for urban planning and water resource protection in the future, and hold great significance for urban sustainable development.

## 1. Introduction

Water is not only the natural resources to maintain the human existence, but also the material basis to guarantee the socio-economic development. Water resources directly or indirectly provide resources guarantee for social development [1]. As the important strategic socio-economic resource for the development of urbanization, the rational and efficient utilization of water resources will affect the implementation of sustainable urban development strategies [2,3]. Urbanization system includes complex relationships of dependence between socio-economic activities and resources. Water resource security and urban development systems are intricately linked [4]. Due to the rapid development of urbanization, urban population has increased dramatically, and the scale of urbanization has been expanding, resulting in increasing pressures on urban water resources [5]. Recently, water scarcity caused by urbanization has brought a series of socio-economic issues. According to statistics, about 2.6 billion people in the world lack access to safe water resource [6]. Clarifying the relationship between urbanization and water resource security (WRS) is not only the basis for effective water resources management, but is also required to achieve a sustainable urban development.

The concept of WRS was first developed in the late 20th century. In a broad sense, WRS refers to a state in which water resources can ensure the healthy and stable existence and development of human beings [7,8]. The term WRS indicates the risks to water supply and demand, and to social and economic development. In our research, WRS means that the function and state of the water environment system on which humans rely should be in a healthy, complete, and stable, and can provide ecological services for human survival and socio-economic development. Previous studies have appreciated that urbanization has a strong interaction with water resources [9,10]. On the one hand, the urbanization process relies heavily on water resource consumption. Water resources are required to ensure the healthy operation of industry, agriculture, and other economic activities [11]. On the other hand, water shortage and water pollution caused by socio-economic development can induce economic losses and threaten the sustainable development of human society [12]. At present, there is evidence that problems related to water scarcity and water pollution are increasing, mainly due to overconsumption of water resources and sewage discharge. It appears that there is a significant unbalance between urbanization and WRS. The need to balance between these two aspects is clear, and is necessary to ensure a sustainable development.

Academic study on the effects of urbanization on the environment have mainly focused on water resources [13], land use [14], and energy consumption [15]. These studies examined the impacts of urban scale, population density, and transportation network on the eco-environment. An increasing number of scholars are concerned about ensuring a harmonious relationship between economic development and the environment, as well as the reasonable utilization of resources [16]. At present, the concept of coupling has been widely used in the fields of ecology and climate change. It mainly means that two or more subjects interact reciprocally in various forms [17]. Environmental assessments have gradually evolved from a single environmental analysis to the exploration of the harmonious relationship among economic development and environment [18,19]. For example, Han et al., [20] analyzed the coupling state between urbanization and water ecosystem from the spatial–temporal perspective. Al-Mulali et al., [21] found an inverted U-shaped coupling relationship between ecological environment and urbanization in both middle- and high-income countries. Yao et al., [22] assessed the spatiotemporal changes in coordinating state of new urbanization and ecological-environmental stress.

Recently, one of the biggest challenges facing society is how to maintain natural resources while promoting social development and quality of human life [23]. Sustainable development theory holds that economic growth should be based on strict population control, improvement population quality, sustainable resources use and environmental protection. This theory regards the coordination state between the resources and urbanization as a key indicator to judge whether the urban development is sustainable [24]. Society should examine the coordination between urbanization development and resources to protect resources, especially water resources. However, there are few studies on the harmonious relationship between WRS and urbanization. Therefore, there is an urgent need to accurately assess the coupling degree of these two systems, as this is crucial for the realization of sustainable urban development. In addition, about the analysis of urbanization, most scholars pay attention to the urban land expansion [25], such as land use conflicts, land use synergies and tradeoffs, and land-use/land-cover change [26]. In fact, the urbanization process includes not only the land use, but also demographic change, economic growth and social development [20], which is the combination process of these four aspects. The coupling coordination model can integrate the four aspects of urbanization with WRS, which contributes to the analysis of sustainable urban development. Thus, it is important to have an adequate understanding of the coordination between urbanization and WRS to support for new-urban planning.

To fill this gap, we attempt to introduce the improved coupling coordination degree (ICCD) model and an obstacle degree model to analyze the coordination relationship between WRS and urbanization and its obstacle factors in Beijing city. We firstly establish an indicator system including the urbanization system and the WRS system. Then, we use the ICCD model to examine the coordination state between urbanization-WRS in Beijing city from 2008 to 2017. Finally, we introduce the obstacle degree model to determine the dominant obstacle indicators. This study will provide new insights into the water resources management and new-urban development in the future.

## 2. Materials and Methods

### 2.1. Study Area

**Figure 1:**
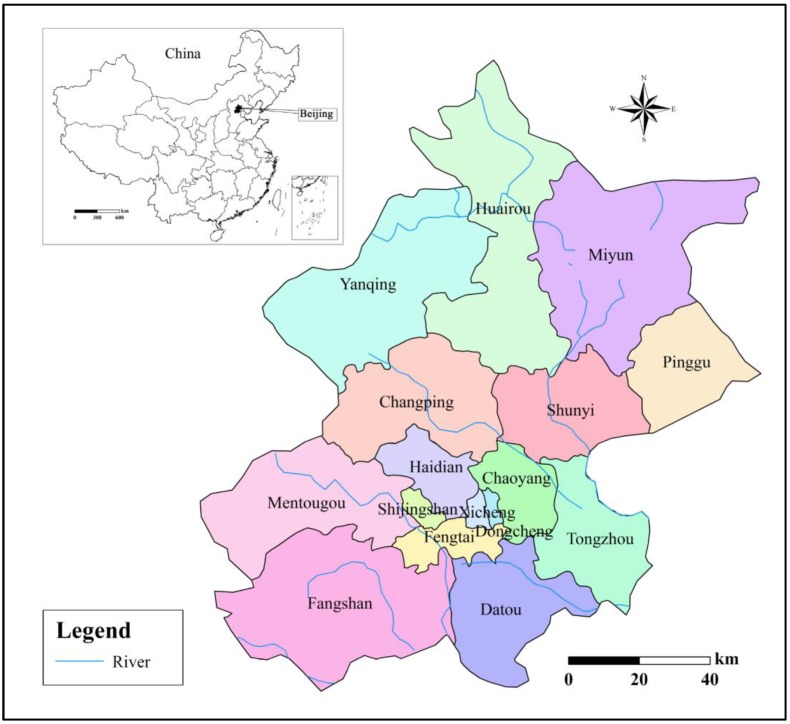
Location of Beijing city.

Beijing city, the capital of China, is located in the north of China. It covers an area of 1.64 million square kilometers, and is divided into 16 major districts (see Figure 1). Beijing lies between 39°26′–41°03′ N and 115°25′–117°30′ E, in a warm, temperate, with moderate warmth and four seasons. Beijing, as one of the fastest growing urbanization areas in China, has a high socio-economic level; however, the gap between urbanization and water resource is very large. As the center of politics, economy, and culture in China, in 2016 the urbanization rate of Beijing reached about 86.5%; the total amount of water resources availability was 3.506 billion cubic meters, while the water resource amount per capita was only 161 billion cubic meters, i.e., about 1/15 of the national average [27,28]. Water resource security in Beijing is a quite prominent problem, especially water supply and demand, and seriously influences the ecological health and the socio-economic development.

### 2.2. Methods

#### 2.2.1. Research Framework

**Figure 2:**
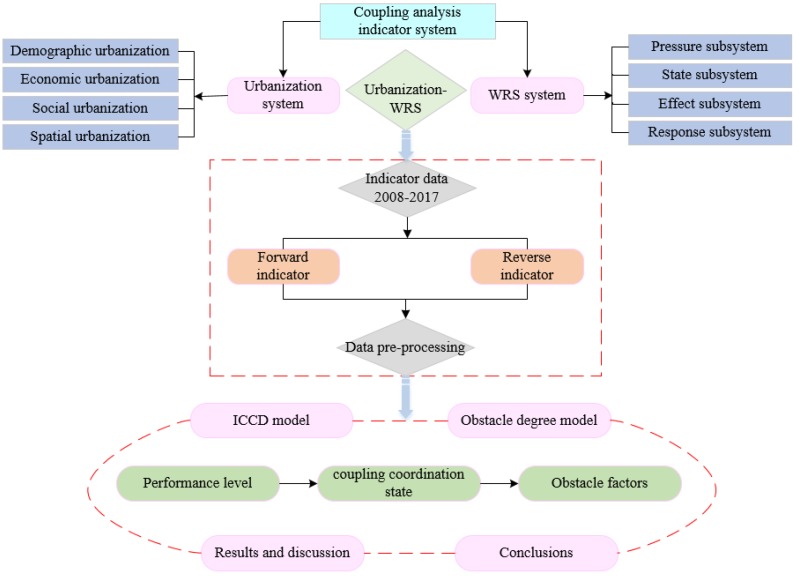
The research framework.

This paper tries to explore the coordination relationship between WRS and urbanization by using a systematic framework (see Figure 2). For this purpose, (1) we establish a comprehensive indicator system, including the urbanization and WRS systems; (2) the data of forward and reverse indicators are preprocessed into dimensionless values; (3) the ICCD model and obstacle degree model are established; and (4) the coupling coordination state between the urbanization-WRS system is analyzed.

#### 2.2.2. Construction of Indicator System

Urbanization is a way to describe the process of continuous concentration of population into urban areas. This is a process of continuous development and change, in which the type of urbanization changes from “traditional” to “new-type”, the social economy is constantly developing, population quality is constantly improving, and urban development moves toward modernization [29]. Urbanization has an important impact on several aspects of society, population, and economic life [30]. Recently, numerous scholars have carried out research on China’s urbanization development from four aspects of demographic urbanization, economic urbanization, social urbanization, and spatial urbanization [31,32]. In this study, we followed this classification to establish the primary indicators for the urbanization system. In terms of the specific secondary indicators, we selected urban population density, non-agricultural population rate, and population growth rate to represent demographic urbanization. These indicators are commonly used as secondary indicators for demographic urbanization [33]. Economic urbanization is the geographical concentration of urban economic activities in the process of urbanization. Thus, the indicators of per capita financial revenue, per capital GDP, and per capita investment in fixed assets can be employed to evaluate economic urbanization. The extent of social urbanization is quite wide; therefore, we chose three representative indicators (i.e., per capita education funds, number of doctors per 10^4^ people, and number of public transports per million people) to represent it, based on existing research [34]. Spatial urbanization mainly shows the changes in land use and fixed assets investment. Dwelling area per capita, urban road area per capita, and fixed asset investment growth rate were selected as indictors of spatial urbanization. Thus, a total of 12 representative indicators were determined for urbanization.

The construction of the WRS indicator system followed the Pressure-State-Effect-Response (PSER) framework, which is widely used in the field of environment and sustainable development [35,36]. The PSER framework includes the pressure subsystem, the state subsystem, the effect subsystem, and the response subsystem, as well as their interactions and constraints. The PSER framework can clearly show the causal relationship between these four WRS subsystems. In terms of secondary indicators, we determined the indicator system of WRS through 4 primary indicators and 14 secondary representative indicators based on the status quo of the water resources and the water environment in Beijing [20,37].

**Figure 3:**
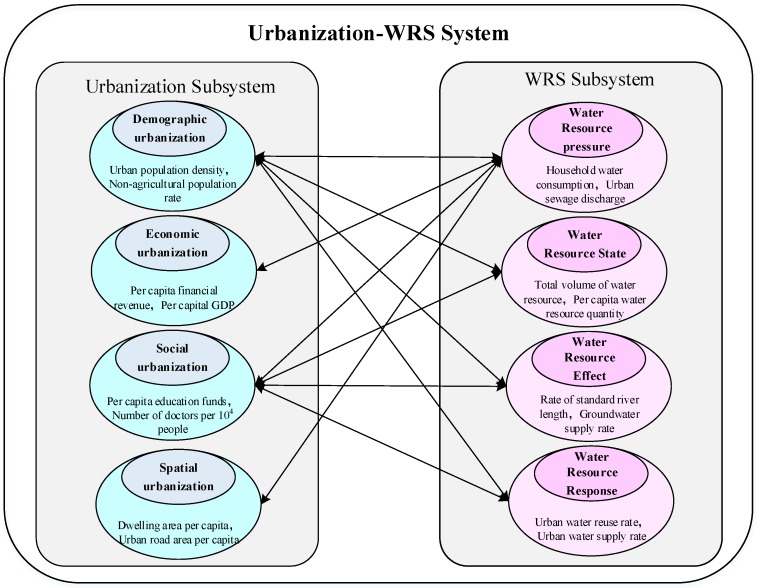
Urbanization-WRS system connotation structure model.

In general, the urbanization system and WRS system couple with each other and have complex interactions. We selected the 12 representative urbanization indicators and 14 WRS indicators to reflect the relationship between the two systems. The structure model of urbanization-WRS system in Beijing is shown in Figure 3 [38]. The indicator systems for urbanization and WRS are shown in Table 1.

#### 2.2.3. The Variation Coefficient Method

(1) Pre-processing of the indicators.

The WRS indicator system includes both forward and reverse indicators. For the forward indicators (i.e., the state subsystem indicators D5–D6), a larger indicator value means a better performance level. For the reverse indicators (i.e., the pressure subsystem indicators D1–D4), a bigger indicator value means a worse performance level. Thus, it is necessary to convert the indicators to dimensionless values to avoid the effect of the characteristics and the scope of the selected indicator. The following normalization formulas were used:

(1) $$\mathrm{Forward}\;\mathrm{indicator}:\;f_{ij}=(x_{ij}-\min\left\{x_{i}\right\})/(\max\left\{x_{i}\right\}-\min\left\{x_{i}\right\}),$$

(2) $$\mathrm{Reverse}\;\mathrm{indicator}:\;f_{ij}=(\max\left\{x_{i}\right\}-x_{ij})/(\max\left\{x_{i}\right\}-\min\left\{x_{i}\right\}),$$

where *x_ij_* is the original index value and *f_ij_* is the standardized value of *x_ij_*.

(2) Determination of indicators’ weight.

The urbanization and WRS systems cover 12 and 14 indicators, respectively. A way to distinguish the importance of these indicators in each system is to assign them different weights. The variation coefficient method is based on the information contained in the index data to evaluate the importance of difference indexes, which is a method of objectively calculating weights [39,40]. The variation coefficient method believes that when the variation degree of the indicator is greater, the ability of the indicator to distinguish different evaluated objects should then be stronger, and the weight should be larger [41]. The variation coefficient method has been widely introduced to environmental quality assessments [42]. The variation coefficient weight was calculated with the following steps:

(3) $$\bar{x_{i}}=\frac{1}{n}\sum_{j=1}^{n}x_{ij}$$

(4) $$\sigma_{i}=\sqrt{\sum_{j=1}^{n}(x_{ij}-\bar{x_{i}}{)}^{2}/n}$$

(5) $$d_{i}=\sigma_{i}/\bar{x_{i}}$$

(6) $$w_{i}=d_{i}/\sum_{i=1}^{m}d_{i}\;(0<w_{i}<1,\sum_{i=1}^{m}w_{i}=1)$$

where $\bar{x_{i}}$ refers to the average value of *x_i_* during the period studied; *σ_i_* represents the standard deviation of *x_i_*; *d_i_* is the variation coefficient of *x_i_*, it represents the variation degree of *x_i_*; and *w_i_* is the weight of the indicator *x_i_*.

#### 2.2.4. The ICCD Model

The system composed of both WRS and urbanization can be seen as a coupling system, in which WRS and urbanization influence and restrict reciprocally [43]. The degree of interaction among systems is measured by coupling coordination degree [44]. The contribution coefficients describe the influence degree of each single system to the whole coupling system. The ICCD model is shown below [45]:

(1) Determination of the performance levels.

The performance levels of the two systems were calculated in the following way:

(7) $$U_{j}=\sum_{i=1}^{m}w_{i}^{u}\cdot f_{i}^{u}$$

(8) $$W_{j}=\sum_{i=1}^{m}w_{i}^{w}\cdot f_{i}^{w}$$

where *U_j_* is the urbanization system’s performance level; *W_j_* refers to the WRS system’s performance level; *f_i_^u^* and *w_i_^u^* are the standardized value and the weight of the indicator *x_i_* in urbanization system, respectively; and *f_i_^w^* and *w_i_^w^* are the standardized value and the weight of indicator *x_i_* in WRS system, respectively.

(2) Determination of the improved contribution coefficients.

In the ICCD model, it needs to determine the contribution coefficients of urbanization system and WRS system. The traditional CCD model usually utilizes subjective assignment methods to determine the contribution coefficients. The two contribution coefficients are arbitrarily assigned the value of 0.5 by some scholars. The way determines the contribution coefficients by assigning the fixed value (0.5) in a subjective way, which highlights the subjective judgment of the decisionmakers [46,47]. Thus, we introduced an ICCD model to determine the contribution coefficients [48] in the following way:

(9) $$a^{*}=W/(U+W)$$

(10) $$b^{*}=U/(U+W)$$

where $a^{*}$ is improved contribution coefficient of urbanization system; $b^{*}$ is the improved contribution coefficient of WRS system.

(3) Calculation of the coupling coordination degree D.

The D was determined in the following way:

(11) $$C={\left[(U*W)/{\left(\frac{U+W}{2}\right)}^{2}\right]}^{1/k}$$

(12) $$T=a^{*}U+b^{*}W$$

(13) $$D=\sqrt{C\cdot T}$$

where C is the degree of coupling; *k* is the adjustment coefficient, it is usually assigned the value of 2. T is the overall performance level; and D refers to the coupling coordination degree between these two systems.

#### 2.2.5. Obstacle Degree Model

To effectively improve the coordination status of urbanization and WRS, it is necessary to explore the main obstacle factors that have a negative impact on the harmonious relationship between urbanization-WRS system. The main step of obstacle degree model is proposed as follows [49]:

(14) $$Q_{i}=w_{i}\cdot (1-f_{ij})/\sum_{i=1}^{m}w_{i}(1-f_{ij})$$

where $Q_{i}$ represents the obstacle degree, it means the influence degree of each subsystem or indicator on the two systems. $1-f_{ij}$ refers to the deviation degree of the indicator, it represents the difference between the actual indicator value and the optimal target value.

#### 2.2.6. Classification Standard of Coupling Coordination Degree

Previous studies have shown that the states of coupling coordination between urbanization and WRS are divided into five grades, namely serious imbalance, imbalance, basic coordination, coordination, and good coordination [21,32]. Accordingly, this study adopted this classification. The *D* was used to describe the coordination status between two interacting systems, and the five grades were defined as follows:

(1) Serious imbalance state: when *D*^∗^ assumes a value within the range 0 ≤ *D*^∗^ ≤ 0.25. In this case, the nexus between urbanization and WRS is very poor.
(2) Imbalance state: when *D*^∗^ assumes a value within the range 0.25 < *D*^∗^ ≤ 0.45. In this case, the interaction between urbanization and WRS is weak.
(3) Basic coordination state: when *D*^∗^ assumes a value within the range 0.45 < *D*^∗^ ≤ 0.65. In this case, the link between urbanization and WRS begins to reinforce.
(4) Coordination state: when *D*^∗^ assumes a value within the range 0.65 < *D*^∗^ ≤ 0.75. In this case, the relationship between urbanization and WRS is coordinated.
(5) Good coordination state: when *D*^∗^ assumes a value within the range 0.75 < *D*^∗^ ≤ 1. In this case, the coordination between urbanization and WRS is very good.

### 2.3. Data Source

The urbanization system data during the period 2008–2017 were collected from the Beijing Statistical Yearbook [27] and the China Urban Statistical Yearbook [50]. The annual data on the WRS system were collected from the Beijing Water Resource Bulletin [28] and the Beijing Statistic Yearbook on Environment [51].

## 3. Results

### 3.1. Performance Level of Two Systems

**Figure 4:**
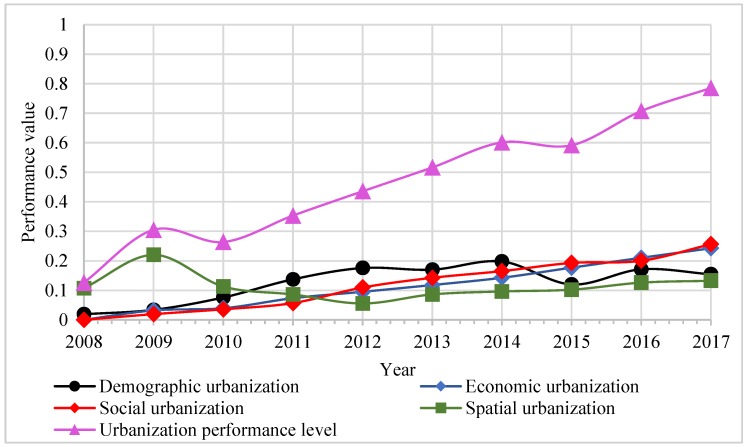
Performance level trends in the urbanization system from 2008 to 2017.

The overall performance level of urbanization displayed an upward trend during the period 2008–2017, as shown in Figure 4. Figure 4 shows that, except for 2010 and 2015, the overall performance level of urbanization has been increasing steadily in the other years, from 0.13 in 2008 to 0.79 in 2017. It is noteworthy that the value in 2017 is five times larger than that in 2008. In terms of the subsystems’ performance level, the performance values of the four urbanization subsystems showed different degrees of increase for each year. Spatial urbanization displayed a clear fluctuation, with its performance value increasing from 0.11 in 2008 to 0.22 in 2009, before declining to 0.06 in 2012, and finally reaching 0.13 in 2017. The other three subsystems showed a steady upward trend during the period studied, with social urbanization recording the highest growth trend, from 0.02 in 2008 to 0.256 in 2017.

**Figure 5:**
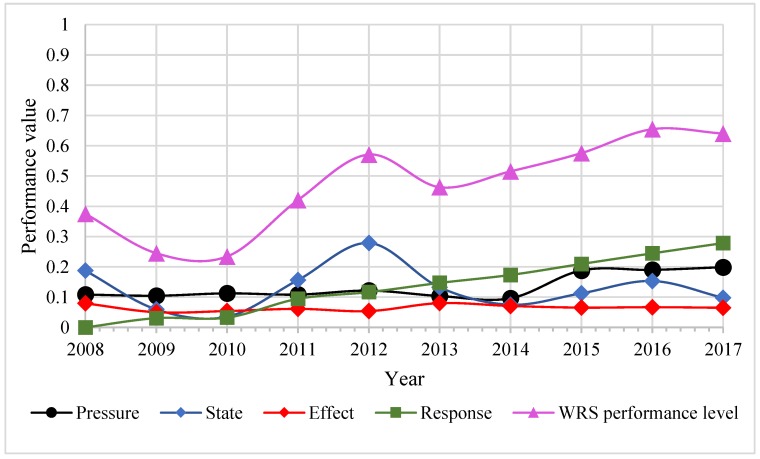
Performance level trends in the WRS system from 2008 to 2017.

Figure 5 shows the trends of the performance level in the WRS system from 2008 to 2017. The overall performance level of WRS followed an S-shaped growth curve, which initially decreased, and subsequently clearly increased from 2008 to 2012. Thereafter, the performance value in the WRS system largely increased from 2014 to 2017. The fluctuation of the performance level in the WRS system was mainly due to the performance level of both the state and the effect subsystems, which declined from 2008 to 2010, but raised continuously after 2010. From 2012 onwards, the two subsystems showed a downward trend of volatility. It is worth noting that the changing trend of the state subsystem was similar to that of the overall performance level. The fluctuation of both the state and the effect subsystems caused the S-shaped growth curve of the overall trend. The performance level of the pressure and response subsystems gradually increased with a significant growth trend from 2014 to 2017.

### 3.2. Coupling Coordination State

**Figure 6:**
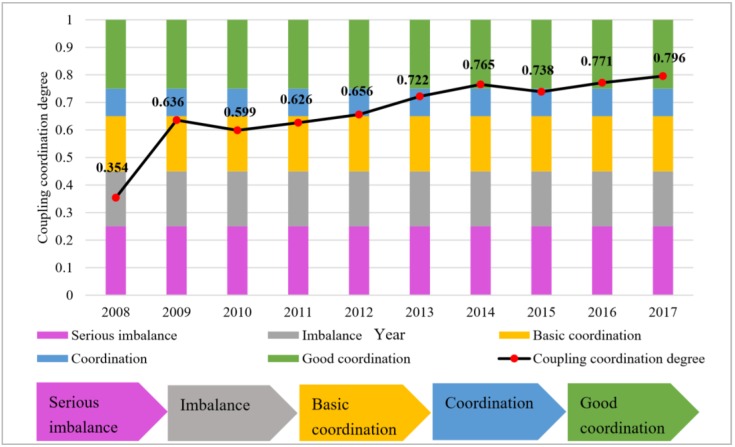
Coupling coordination degree trends from 2008 to 2017.

Figure 6 reveals the results of the coupling coordination degree *D* between urbanization and WRS in Beijing from 2008 to 2017. As shown in Figure 6, the *D* between urbanization and WRS followed an overall rising tendency during the period studied, from 0.354 in 2008 to 0.796 in 2017. The results of *D* mean that the coupling coordination state between the urbanization-WRS system has improved. The state of the coupling coordination between the two systems shows a dynamic evolution, passing from an imbalance state to a good coordination state during the 2008–2017 period. Specifically, by comparing the standard *D*^∗^ with the actual *D*, we find that the two systems in Beijing city was in an imbalance state in 2008. During the 2009–2012 period, the coordination between the two system in Beijing maintained in a basic coordination state. In the following four years, D values between urbanization-WRS in Beijing were basically in a good coordination state.

### 3.3. Dominant Obstacle Factors

Using the obstacle degree model, the obstacle degrees of the subsystem in the urbanization and WRS systems were obtained; they are shown in Table 2. As shown in Table 2, in the urbanization system, the subsystem with the greatest obstacle degree was social urbanization during 2008 to 2010; the subsystem of spatial urbanization had the largest obstacle degree from 2011 to 2017. In comparison, the demographic urbanization subsystem had the lowest obstacle degree during the 2008–2017 period. This means that in Beijing city, both the social and spatial urbanizations were the domain obstacle subsystems, while the demographic urbanization was the least important subsystem. In the WRS system, the pressure subsystem had the greatest obstacle degree in the first six years during the surveyed period and the state subsystem had the biggest obstacle in terms of degree in the remaining four years, whereas the effect subsystem had the smallest during the period 2008–2017. These findings suggest that, both the pressure and state subsystems were considered to have the greatest hindrance to the development of urbanization-WRS system. On the contrary, the effect subsystem had the smallest impact on the coordination state between urbanization-WRS system in Beijing city.

In urbanization system, the top five indicators that hinder the coordination development of urbanization-WRS system are shown in Table 3. From 2008 to 2010, the indicators of *C12* fixed asset investment growth rate, *C9* number of public transports per million people, and *C10* dwelling area per capita were ranked in the top three in the urbanization system. After 2010, the indicator of *C11* urban road area per capita replaced the *C10* dwelling area per capita became one of the top three indicators. In terms of the WRS system, the indicators of *D1* water consumption for industrial production, *D2* water consumption of agricultural irrigation and *D14* urban water supply rate ranked as the top three indicators from 2008 to 2017. This means that the three indicators had a profound effect on the coordination state between urbanization and WRS.

## 4. Discussion

### 4.1. Analysis of Performance Level

The results of the urbanization system’s performance level suggest that in the past decade, the performance level of urbanization system in Beijing city has improved. During the four subsystems in urbanization, social urbanization is considered to make the greatest contribution for the whole performance level. In the past 10 years, Beijing city was undergoing a period of urbanization and industrialization, relying on its location advantage and policy support, thus achieving a fast increase in the social urbanization level. In relation to WRS, the overall performance levels showed a fluctuating upward trend. The performance levels of the pressure and response subsystems were constantly optimized. This is mainly due to the fact that in 2016, Beijing was listed as the second demonstration “Sponge City”. The concept of “Sponge City” is new in urban water management; it aims to effectively divert rain and sewage, store rainwater, and purify sewage. In Beijing city, the response measures adopted in the context of the “Sponge City” released the pressures on water resources, thereby contributing to the improvement of the overall performance levels in the WRS system.

### 4.2. Analysis of Coupling Coordination State

**Figure 7:**
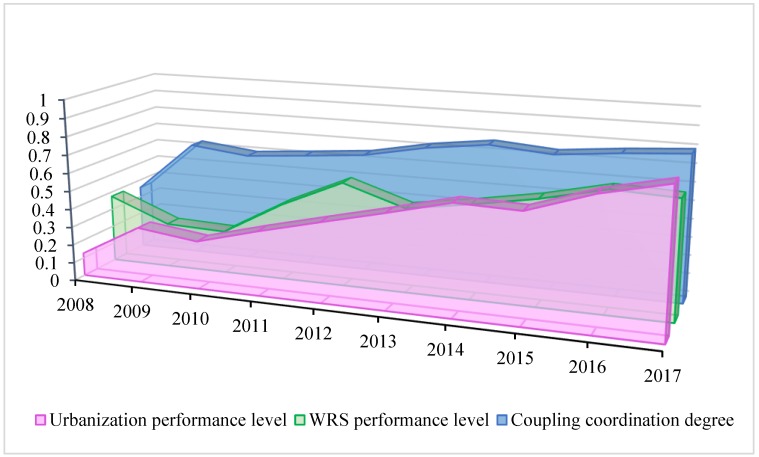
Trend comparison of urbanization performance level and WRS performance level.

We examined the coordination state between the WRS and urbanization systems by analyzing the performance gap between the urbanization and the WRS systems. The trend comparison of urbanization performance level and WRS performance level is shown in Figure 7. We discussed this dynamic evolution from the following three stages (2008–2010, 2010–2014, and 2014–2017).

(1) 2008–2010: The *D* between urbanization and WRS was low, and grew rapidly. The nexus between urbanization and WRS was initially weak, and gradually began to strengthen. In 2008, the performance level of urbanization was poor, while the performance level of WRS was considerably higher. As a result, an unbalanced state occurs between the two systems in Beijing. Subsequently, as the gap between urbanization performance level and WRS performance level decreases gradually, while the *D* among urbanization and WRS grew more rapidly, increasing from 0.354 in 2008 to 0.636 in 2009. Therefore, the coupling coordination state was optimized. During the period from 2009 to 2010, the impact of urbanization on WRS strengthened, and the performance level of WRS was at its lowest point. As a result, the *D* showed a slight decline. Fortunately, this decline did not cause changes in the coupling coordination state. Both in 2009 and 2010, the urbanization-WRS system was in a basic coordination state.

(2) 2010–2014: The *D* between urbanization and WRS raised steadily. During this period, urbanization in Beijing entered a stage of rapid development, and the WRS state improved due to the implementation of the South-to-North Water Diversion Project in 2012. The levels of urbanization and WRS increased simultaneously. The gap in the performance levels between the two systems was reduced, signaling their simultaneous development. In this period, the *D* between the two systems increased from 0.599 in 2010 to 0.765 in 2014. Therefore, the coupling coordination state improved greatly, displaying a cross-level change. The coupling coordination state moved from a basic coordination state in 2010–2011, to a coordination state in 2012–2013, and eventually to a good coordination state in 2014. This finding demonstrates that these processes were gradually coordinated during this period.

(3) 2014–2017: The *D* between urbanization and WRS slowly increased. After the implementation of the “new-type urbanization” plan in 2014, Beijing has experienced a rapid urbanization process. The “new-type urbanization” is a new concept of urban development, based on the tenets of resource conservation, eco-friendliness, and sustainable development. During this period, a series of new-type urbanization measures were taken, such as the construction of the “Sponge City”, the protection of the eco-environment, and the renovation of the underground pipeline network, which contributed to the improved quality urbanization and water environment. Not only the urbanization level, but also water resource quality was constantly enhanced. The urbanization and WRS systems were in a good coordination state during the period 2016–2017. Urbanization went in a coordinated development phase with water environmental protection. The coupling coordination state among WRS and urbanization improved slowly.

### 4.3. Analysis of Obstacle Factors

In the urbanization system, both social and spatial urbanizations had the greatest influence on coordination development of urbanization-WRS in Beijing city, while demographic urbanization had the smallest impact. Thus, social and spatial urbanizations subsystems need to be considered by policy-makers wishing to improve the coordination development of the two systems. In parallel, the pressure and state subsystems played a dominant role in the WRS system, while the effect subsystem had a minimal effect. The research results are consistent with the current situation of WRS in Beijing. They suggest that, the development of socio-economy has brought tremendous pressure on water resources in Beijing over the past ten years. The state of WRS hinder the development of two systems. Given these circumstances, it is necessary to take more reasonable water resource conservation measures to release the stress on water resources.

In relation to the secondary indicators, the statistical results of obstacle factors show that fixed asset investment growth rate, number of public transports per million people, dwelling area per capita and urban road area per capita were the key indicators restricting the healthy development of urban and water resources. These findings show that, during the 2008–2017 period, the urbanization population has increased rapidly, and Beijing’s infrastructure is insufficient to meet the needs of the increased urban population. Water consumption for industrial production, water consumption of agricultural irrigation and urban water supply rate were the three key indicators that had the highest influence in the health development of two systems. Beijing is located in the north of China, in an area with a dry climate; this has led in the past to serious shortages of water resources. Since the reform and opening-up of the economy, Beijing is experiencing a process of industrialization and urbanization, and the industrial and agricultural development has led to an increase in industrial and agricultural water use which pose a significant risk to water resources security.

## 5. Conclusions

This study explored the coordination relationship between urbanization and WRS in Beijing by using the ICCD model and obstacle degree model. The main contributions of this research are as follows. Firstly, the indicator systems of urbanization and WRS have been established. These indicators can provide criteria to measure changes in the coupling degree in both systems. Secondly, this study revealed the dynamic trends present in the coupling coordination state between urbanization and WRS, using the ICCD model. Thirdly, the indicators that restrict the coordination development of urbanization and WRS systems are determined by using the obstacle degree method, which can provide a solid knowledge base for policy-makers to adjust water resources use plan and urbanization development policies.

Over the past ten years, Beijing has experienced a rapid urbanization process, which put a lot of pressure on water resources security. Overall, the state of the coupling coordination between the two systems has improved, passing from an imbalance state towards a good coordination state. However, the coupling coordination degree between the two systems is still low. The social urbanization, spatial urbanization, pressure and state subsystems are the main obstacle subsystems.

According to the research results, we propose the following suggestions and prospects: (1) policy-makers should adjust the urban development strategy by changing the development mode of spatial urbanization. The “chain effect” of medical, education, and infrastructure development on social urbanization can be improved through a rational allocation of resources. Moreover, more attention should be paid to the environmental factors when formulating the new-urbanization development strategy; (2) In order to guarantee the sustainable development of urbanization and water resources, the government should pay more attention on relieving the pressures on water resources caused by the rapid urban development. The local government needs to increase the investment to optimize the industrial structure, promote the adoption of innovative water-saving technologies, and develop clean energy to reduce sewage discharge. In addition, it is necessary to propose a water consumption strategic plan to improve water utilization effect and reduce water resource consumption of industrial output.

The coordination relationship between urbanization and WRS is a very complicated system. Due to data source limitations, this study focused only on the investigation of one city, i.e., Beijing. Future research should focus on the methodology to perform a comprehensive analysis of China’s urban agglomerations, such as the Beijing-Tianjin-Hebei urban group. A comparative analysis should be performed of different regions in the urban group through the use of spatial gradients and temporal scales, to have a win-win effect.

## Figures and Tables

**Table 1:** Urbanization-WRS indicator system.

System	Subsystem	Indicators	Unit	Type
Urbanization	Demographic urbanization	*C_1_* Urban population density	Person/km^2^	+
		*C_2_* Non-agricultural population rate	%	+
		*C_3_* Population growth rate	‰	+
	Economic urbanization	*C_4_* Per capita financial revenue	10^4^ Yuan/person	+
		*C_5_* Per capital GDP	10^4^ Yuan/person	+
		*C_6_* Per capita investment in fixed assets	10^4^ Yuan/person	+
	Social urbanization	*C_7_* Per capita education funds	Yuan/person	+
		*C_8_* Number of doctors per 10^4^ people	/	+
		*C_9_* Number of public transports per million people	/	+
	Spatial urbanization	*C_10_* Dwelling area per capita	m^2^/person	+
		*C_11_* Urban road area per capita	km^2^/person	+
		*C_12_* Fixed asset investment growth rate	%	+
WRS	Pressure	*D_1_* Water consumption for industrial production	10^9^ m^3^	—
		*D_2_* Water consumption of agricultural irrigation	10^9^ m^3^	—
		*D_3_* Household water consumption	10^9^ m^3^	—
		*D_4_* Urban sewage discharge	10^9^ m^3^	—
	State	*D_5_* Total volume of water resource	10^9^ m^3^	+
		*D_6_* Per capita water resource quantity	m^3^/person	+
	Effect	*D_7_* Water producing coefficient	%	+
		*D_8_* COD emissions	10^4^ tons	—
		*D_9_* Rate of standard river length	%	+
		*D_10_* Groundwater supply rate	%	+
	Response	*D_11_* Rate of water resources management investment to GDP	%	+
		*D_12_* Rate of industrial wastewater up to discharge standard (RIWDS)	%	+
		*D_13_* Urban water reuse rate	%	+
		*D_14_* Urban water supply rate	%	+

**Table 2:** Obstacle degree of subsystem in the two systems.

System	Urbanization				WRS			
Year	Demographic Urbanization	Economic Urbanization	Social Urbanization	Spatial Urbanization	Pressure	State	Effect	Response
2008	21.2%	27.8%	29.3%	21.7%	45.4%	15.9%	6.9%	31.8%
2009	11%	30.3%	34.1%	24.5%	33.5%	30.1%	9.6%	26.8%
2010	17.5%	27.6%	29.9%	25%	32.8%	25.4%	9.0%	32.8%
2011	10.4%	26.2%	30.8%	32.6%	34.3%	22.5%	10.6%	32.6%
2012	5.1%	26.1%	26.0%	42.8%	43.3%	16.0%	1.8%	38.9%
2013	7.1%	25.8%	23.5%	43.6%	37.9%	28.8%	7.9%	25.4%
2014	1.7%	25.1%	22.9%	50.3%	36.1%	36.2%	8.9%	18.8%
2015	15.6%	16.1%	20.7%	47.7%	28.1%	41.0%	13.5%	17.4%
2016	11.1%	11.3%	19.2%	58.4%	34.0%	38.6%	11.3%	16%
2017	0.0%	23.3%	0.1%	76.6%	30.1%	52.4%	1.4%	16%

**Table 3:** The dominant obstacle indicators in the two systems.

Urbanization						WRS				
Indicator Order	1	2	3	4	5	1	2	3	4	5
2008	C12	C9	C10	C8	C4	D1	D14	D2	D8	D11
2009	C9	C12	C10	C8	C4	D1	D14	D2	D8	D5
2010	C9	C10	C12	C8	C4	D1	D14	D2	D8	D5
2011	C11	C9	C12	C8	C7	D1	D14	D2	D12	D9
2012	C11	C12	C9	C10	C4	D1	D14	D9	D2	D12
2013	C11	C12	C9	C4	C8	D1	D14	D2	D6	D5
2014	C12	C11	C4	C8	C10	D1	D2	D14	D7	D5
2015	C12	C11	C9	C3	C3	D14	D2	D1	D10	D5
2016	C12	C11	C9	C3	C7	D6	D14	D1	D10	D5
2017	C12	C11	C3	C2	C1	D14	D1	D2	D10	D4
